# Effects of supplementation with rumen-protected methionine on growth, feed efficiency, blood parameters, and health of weaned Holstein growing calves

**DOI:** 10.5455/javar.2025.l979

**Published:** 2025-12-25

**Authors:** Hamed Nikookalm, Farhad Foroudi, Naser Karimi, Ghobad Asgari, Kazem Karimi, Ali Afsar

**Affiliations:** 1 Department of Animal Science, Varamin-Pishva Branch, Islamic Azad University, Varamin, Iran; 2 Nutrition Department, Technical Manager, Evonik Co. Iranian Branch, Tehran, Iran

**Keywords:** Blood parameters, feed efficiency, gain, Holstein calf, rumen-protected methionine

## Abstract

**Objective**: This study evaluated the effects of dietary supplementation with rumen-protected methionine (RPM) on growth performance, feed efficiency (FE), blood parameters, and health status in post-weaning calves.

**Materials and Methods**: Forty weaned calves were randomly allocated to four groups: G1. No RPM supplementation (control); G2. RPM supplementation for 10 days post-weaning (days 75–85); G3. RPM supplementation for 20 days post-weaning (days 75–95); G4. RPM supplementation for 30 days post-weaning (days 75–105). Ethyl cellulose-coated D-L methionine was administered at 2 gm/day/calf. Growth performance, FE, serum biochemical parameters, complete blood count (CBC), and health indicators were investigated.

**Results**: G4 showed the highest weight gain (1.01 kg/day) and FE (0.54), as well as the lowest feed conversion ratio (FCR) (1.84) (*p <* 0.05). In contrast, G1 had the lowest weight gain (0.82 kg/day) and FE (0.51), along with the highest FCR (1.98) (*p <* 0.05). RPM supplementation significantly reduced blood urea nitrogen, aspartate aminotransferase, and alanine aminotransferase levels (*p <* 0.05) but had no significant effect on total protein, triglyceride, or cholesterol levels. Additionally, it increased RBC count and hemoglobin concentration (*p <* 0.05), while WBC counts, lymphocytes, and platelets remained unaffected. Moreover, RPM supplementation significantly improved overall health and reduced the incidence of diarrhea (*p <* 0.05).

**Conclusion**: Dietary supplementation with RPM had a positive influence on growth performance, FE, blood parameters, and overall health in growing calves. A daily dose of 2 gm/day/calf is recommended.

## Introduction

The weaning process of calves is considered one of the most sensitive periods of dairy cattle breeding [1–3]. Indeed, optimal nutrition is crucial for maximizing the growth of the calf and the development of its digestive system [4,5]. More specifically, the primary challenge for post-weaning calves is to achieve proper rumen fermentation activity as quickly as possible, thereby taking up nutrients and producing the fatty acids and microbial protein necessary for growth [3-7]. From an economic perspective, a prolonged period of liquid feeding is not cost-effective and negatively impacts the profitability of the animal husbandry unit [8,9].

To maximize the growth of weaned calves, the best possible use of solids in the form of fodder and concentrates should be considered as soon as possible [2–4]. Concentrates, which comprise the bulk of a calf’s solid diet, must contain adequate amounts of carbohydrates, protein (including essential amino acids such as methionine and lysine), fat, vitamins, and minerals [3-7]. These amino acids play a crucial role in supporting growth, feed efficiency (FE), and energy utilization [7-9], while also enhancing immunity [5], regulating blood metabolites and liver biomarkers [7,10], and modulating inflammation and oxidative stress [10,11].

Previous studies evaluating the effects of rumen-protected methionine (RPM) supplementation have demonstrated multiple benefits, including improved metabolic profiles [2,7,12], enhanced immune function [5,13,14], and better oxidative stress management [10,14], as well as increased red blood cell (RBC) count and oxygen delivery [15,16]. Furthermore, these benefits contribute to enhanced productive and reproductive performance across various species [12,17-20].

Since methionine and lysine are the first limiting amino acids in microbial protein synthesis [18,21,22], supplementing rapidly growing calves with rumen-protected amino acids has been shown to improve FE and overall health [21-23]. Protecting amino acids from rumen degradation ensures their direct availability for absorption in the small intestine. However, there is a significant knowledge gap concerning the specific effects of coated methionine supplementation on fast-growing calves with underdeveloped rumens. Additionally, limited research has been conducted on its impact during the critical weaning phase, and the interactions between coated methionine and other dietary components in young calves remain poorly understood. Further investigation is warranted to optimize post-weaning nutrition and enhance calf health and productivity.

The objective of this study was to investigate the effects of a diet supplemented with RPM (coated with pH-sensitive polymer) on the growth performance, FE, serum biochemical parameters, blood profile, and overall health of growing calves.

## Materials and Methods

### Ethical approval

All procedures used on the animals in this study were approved by the Islamic Azad University, Institutional Animal Care and Use Committee (Approval No.: IAUEC 2021/1401-425-33-11).

### Animals and experimental design

This experiment was conducted over 30 days (from day 75 to day 105) on a commercial dairy farm (Quarchak Agriculture and Industry Co., Varamin, Tehran, Iran) between March and May 2021 alongside a herd of 2,550 lactating cows. Forty female calves at weaning age (73 ± 2.0 days) with an initial body weight of 89.83 ± 3.42 kg were included in the study*.* The thermal humidity index during the experiment ranged from 71 to 83. After calving, cows were housed in individual calving stalls for 48 h before being transferred to postpartum stalls for 105 days. Calves were randomly assigned to individual pens (1.8 × 2.0 m) with straw bedding and maintained under standard hygienic conditions. Clean drinking water and alfalfa hay were available ad libitum throughout the trial. Calves were fed a total mixed ration twice daily at 06:00 h and 14:00 h. The diet was formulated to meet the National Research Council guidelines [4] (Table 1). RPM (ethyl cellulose-coated D, L-methionine; Mepron^®^, 85% purity, Evonik Industries) was supplemented at 2 gm/day/calf (0.058% DM, based on preliminary trials) via top-dressing. RPM (pH-sensitive polymer-coated D, L-methionine; product code 3c301, 550 gm/kg, packed in 25 kg bags) was homogenized with the feed before being provided to each group. Calves were randomly allocated to four treatment groups from days 75 to 105 as follows: G1: control group receiving only the basal diet (*n* = 10); G2: basal diet supplemented with 2 gm/day RPM for 10 days (days 75–85; *n* = 10); G3: basal diet supplemented with 2 gm/day RPM for 20 days (days 75–95; *n* = 10); and G4: basal diet supplemented with 2 gm/day RPM for 30 days (days 75–105; *n* = 10). RPM (RPM; Mepron^®^, 85% DL-methionine, Evonik Operations GmbH) was top-dressed onto the complete feed.

**Table 1.** Ingredients and chemical composition of the experimental diet.

Ingredients	Percentage (gm/kg of DM unless otherwise noted)
Ground barley	118
Ground corn	455
Soybean meal 44%	255
Corn gluten meal 60%	30
Wheat bran	100
Dicalcium phosphate	6
Edible salt	6
Sodium bicarbonate	10
Vitamin-mineral premix^1^	20
Rumen-protected methionine^2^ (as top-dress)	2
Chemical composition (gm/kg of DM unless otherwise noted)	
CP	216.60
ME^3^ (Mcal/kg DM)	2.71
NEg^4^ (Mcal/kg DM)	1.36
NDF^5^	162
ADF^6^	62.30
Ash	87.30
EE^7^	27.20
NFC^8^	50.69
Lys	9.69
Met	2.61
Lys:Met	3.71

^1^Contained per kilogram of vitamin supplement: 250,000 IU of vitamin A, 50,000 IU of vitamin D, 1,500 IU of vitamin E. Additionally, contained per kilogram of mineral supplement, 2.25 gm of Mn, 120 gm of Ca, 7.7 gm of Zn, 20 gm of P, 20.5 gm of Mg, 186 gm of Na, 1.25 gm of Fe, 3 gm of S, 14 mg of Co, 1.25 gm of Cu, 56 mg of I, and 10 mg of Se.

^2^Rumen protected methionine (D-L methionine-Mepron^®^) mixture.

^3^Mtabolizable energy.

^4^Net energy for growth.

^5^Neutral detergent fiber.

^6^Acid detergent fiber.

^7^EE: Ether extract.

^8^Nonfiber carbohydrate (NFC) = 100–(CP+NDF+Ash+EE).

### Weight records and blood analyses

The initial and final body weights (kg) of the calves were recorded. Feed conversion ratio (FCR) was calculated as the ratio of total feed intake (kg) to body weight gain (kg) (FCR = feed intake/gain). FE was determined as the ratio of body weight gain (kg) to total feed intake (kg) (FE = gain/feed intake). Growth efficiency (Kleiber ratio) was assessed using the ratio of daily weight gain (kg/day) to metabolic body weight (body mass 0.75). One aliquot was analyzed for complete blood count (CBC) parameters, including RBCs, hemoglobin (HGB), white blood cells (WBCs), lymphocytes (Lyms), and platelets (PLTs), using a hematology analyzer (Sysmex XP100) with hemocytometry [24,25]. A second aliquot was centrifuged (3000 × gm, 15 min) to obtain serum, which was stored at −20°C for subsequent biochemical analysis. Serum biochemical analyses included total protein (TP), blood urea nitrogen (BUN), cholesterol (Chol), and triglycerides (TG). Aspartate aminotransferase (AST) and alanine aminotransferase (ALT) activities were determined using commercial kits (Pars Azmoon, Tehran, Iran) on an autoanalyzer (Roche Hitachi-911) according to the manufacturer’s protocols. All measurementswere performed in duplicate. Before analysis, the analyzer was calibrated using control sera (TrueLabN^®^ and TrueLabP^®^) and standard solution (TrueCal U^®^, all from Pars Azmoon, Tehran, Iran).

#### Feed analyses

The concentrate was produced in a single batch as a basal mixture, and a representative sample was collected for chemical analysis. Feed samples were ground through a 1-mm screen using a Wiley mill (Arthur Thomas Co., Philadelphia, PA, USA) before analysis. The dry matter (DM) content was determined by oven-drying samples at 65°C to a constant weight (AOAC method 930.15). Crude protein (CP; N × 6.25) was quantified via Kjeldahl titration (Kjeltec 1030 Auto Analyzer, Tecator, Höganäs, Sweden; AOAC method 920.53). Ether extract (EE) and ash contents were analyzed following AOAC methods 920.39 and 941.12, respectively. Neutral detergent fiber (NDF) and acid detergent fiber (ADF) were determined according to Van Soest et al. [25] using heat-stable α-amylase (100 µl/0.5 gm sample) and sodium sulfite. All analyses were performed in duplicate following AOAC guidelines [24].

#### Statistical analysis

All data were analyzed using SAS 9.2 software (SAS Institute Inc., Cary, NC, USA) in a completely randomized design with repeated measures. Continuous variables (including weight measurements, feed intake data, and blood metabolite concentrations) were evaluated using two-way analysis of variance with the MIXED procedure. The statistical model included calf initial body weight as a covariate to control for its potential effects. Normality of residuals was verified using the Shapiro-Wilk test. Treatment means were compared using Duncan’s multiple range test at a significance level of *p* ≤ 0.05.

## Results

### Growth, feed intake, and performance

Table 2 presents the effects of experimental treatments on feed intake, nutrient utilization, growth performance, and FE parameters in growing calves. The results revealed significantly greater (*p <* 0.05) DM intake (DMI), CP, and net energy consumption, and average daily gain (ADG) in RPM-supplemented groups (G3 and G4) compared to control (G1) and short-term supplemented (G2) groups. Notably, calves in G3 and G4 demonstrated superior FE metrics, including higher Kleiber ratios and improved FCRs (FCR; *p <* 0.05). Among all treatments, G4 achieved optimal performance with maximum ADG (1.01 kg/day) and minimal FCR (1.84), while G1 showed the lowest growth rate (0.82 kg/day) and least efficient FCR (1.98). These findings demonstrate that extended RPM supplementation (20–30 days) significantly enhances growth performance and nutritional efficiency in post-weaning calves.

**Table 2.** Effects of the experimental groups on the feed intake, weight gain and FE indices of growing calves.

Item	Experimental groups ^1^				SEM	*p* -value
	G1	G2	G3	G4		
Initial body weight (kg)	89.47	89.12	88.95	89.21	0.966	0.112
Final body weight (kg)	114.07^b^	115.22^b^	118.35^a^	119.51^a^	0.535	0.005
Feed intake (kg/day)	1.62^b^	1.68^b^	1.82^a^	1.86^a^	0.088	0.006
Protein intake (kg/day)	0.35^b^	0.36^b^	0.39^a^	0.40^a^	0.025	0.011
Net energy intake (Mcal/day)	2.20^b^	2.28^b^	2.48^a^	2.53^a^	0.105	0.005
Gain (kg/day)	0.82^b^	0.87^b^	0.98^a^	1.01^a^	0.056	0.037
FCR^2^	1.98^a^	1.93^a^	1.86^b^	1.84^b^	0.051	0.004
FE^3^	0.51^b^	0.52^ab^	0.54^a^	0.54^a^	0.015	0.044
Kleiber ratio^4^	0.024^c^	0.025^b^	0.027^a^	0.028^a^	0.001	0.001

^a,b,c,^Different superscript letters on the values indicate a significant difference in means at *p* ≤ 0.05.

^1^G1: basal diet, G2: basal diet + 2 gm/day RPM for 10 days, G3: basal diet + 2 gm/day RPM for 20 days and G4: basal diet + 2 gm/day RPM for 30 days.

^2^Feed conversion rate = feed intake/gain.

^3^FE = Gain/feed intake. ^4^This ratio, defined as the growth rate/body mass0, using for measuring growth efficiency. SEM=Standard error of the mean.

### Serum biochemical parameters

Table 3 summarizes the treatment effects on blood biochemical parameters in growing calves. RPM supplementation significantly reduced BUN concentrations across all treatment groups compared to the control (G1), with the most pronounced reduction observed in G4 (*p <* 0.05). However, serum concentrations of TP, TG, and Chol remained unaffected by dietary treatments.

**Table 3.** Effects of the experimental groups on the serum biochemical parameters of growing calves.

Item	Experimental groups ^1^				SEM	*p* -value
	G1	G2	G3	G4		
BUN^2^ (mg/dl)	19.35^a^	17.10^b^	16.55^bc^	16.03^c^	0.340	0.022
TP^3^ (mg/dl)	5.70	5.82	5.78	5.86	0.130	0.555
TG^4^ mg/dl	39.65	40.22	40.30	39.89	1.012	0.704
Chol^5^ mg/dl	76.14	77.23	76.87	78.01	1.896	0.322

^a,b,c,^Different superscript letters on the values indicate a significant difference in means at *p* ≤ 0.05.

^1^G1: basal diet, G2: basal diet + 2 gm/day RPM for 10 days, G3: basal diet + 2 gm/day RPM for 20 days and G4: basal diet + 2 gm/day RPM for 30 days.

^2^Blood urine nitrogen.

^3^Total protein.

^4^Triglyceride.

^5^Cholestrol.

### Serum hepatic enzymes

Table 4 presents the effects of dietary treatments on serum hepatic enzyme activity in growing calves. Significant treatment differences were observed for both AST and ALT concentrations (*p <* 0.05). RPM supplementation significantly reduced serum levels of these hepatic enzymes compared to control groups (*p <* 0.05).

**Table 4.** Effects of the experimental groups on the serum hepatic enzymes of growing calves.

Item	Experimental groups ^1^				SEM	*p* -value
	G1	G2	G3	G4		
AST^1^ (IU/l)	144.86^a^	120.31^b^	94.96^c^	78.86^d^	3.520	0.011
ALT^2^ (IU/l)	41.67^a^	30.35^b^	28.52^b^	28.72^b^	0.650	0.002

^a,b,c,^Different superscript letters on the values indicate a significant difference in means at *p* ≤ 0.05.

^1^G1: basal diet, G2: basal diet + 2 gm/day RPM for 10 days, G3: basal diet + 2 gm/day RPM for 20 days and G4: basal diet + 2 gm/day RPM for 30 days.

^1^Aspartat aminotransferase.

^2^Alanine aminotransferase.

### Hematology and complete blood count

Table 5 demonstrates the treatment effects on complete blood count (CBC) parameters in growing calves. Significant treatment differences were observed for RBC count and HGB concentration (*p <* 0.05), with the highest values recorded in G3 and G4 groups. In contrast, WBC count, Lym percentage, and PLT count remained unaffected by dietary treatments.

**Table 5.** Effects of the experimental groups on the hematology and complete blood count of growing calves.

Item	Experimental groups ^1^				SEM	*p* -value
	G1	G2	G3	G4		
RBC^2^ (Cell/µl)	7.08^b^	7.04^b^	8.11^a^	8.12^a^	0.111	0.021
HGB^3^ (mg/dl)	9.95b	10.15^b^	10.63^a^	10.45^a^	0.190	0.044
WBC^4^ (Cell/µl)	12.07	11.99	12.09	13.00	0.321	0.355
Lym^5^ (Cell/µl)	8.11	7.96	8.08	8.15	0.154	0.401
PLT^6^ (Cell/µl)	189.62	187.36	190.04	191.22	3.550	0.552

^a,b,c,^Different superscript letters on the values indicate a significant difference in means at *p* ≤ 0.05.

^1^G1: basal diet, G2: basal diet + 2 gm/day RPM for 10 days, G3: basal diet + 2 gm/day RPM for 20 days and G4: basal diet + 2 gm/day RPM for 30 days.

^2^Red blood cells.

^3^Hemoglubin.

^4^White blood cells.

^5^Lymphocyte.

^6^Platelet.

### Health characteristics

Table 6 displays the impact of experimental treatments on health parameters in growing calves. Dietary supplementation had a significant effect on the incidence of diarrhea (*p <* 0.05). However, no treatment effects were observed for other health indicators, including pneumonia occurrence, nasal discharge (rhinorrhea), febrile status (fever), general weakness, fecal status, or overall health scores.

**Table 6.** Effects of the experimental groups on the health characteristics of growing calves.

Parameters/Frequency^2^ (%)	Experimental groups ^1^				SEM	*p* -value
	G1	G2	G3	G4		
Diarrhea	30.00^a^	20.00^b^	10.00^c^	10.00^c^	1.13	0.021
Pneumonia	10.00	10.00	ND	ND	1.19	0.455
Rhinorrhea	10.00	10.00	10.00	10.00	1.19	0.355
Febrile status	ND	ND	ND	ND	-	-
General weakness	10.00	10.00	ND	ND	1.10	0.698
Fecal condition score^3^	2	2	2	2	-	-
Overall health score^4^	187	189	190	191	3.550	0.552

^a,b,c,^Different superscript letters on the values indicate a significant difference in means at *p* ≤ 0.05.

^1^G1: basal diet, G2: basal diet + 2 gm/day RPM for 10 days, G3: basal diet + 2 gm/day RPM for 20 days and G4: basal diet + 2 gm/day RPM for 30 days.

^2^Frequency reported based on corrected percentage of animals which showed signs of abnormality calculated by ARCSin√X formula.

^3^Including; 1: hard and firm, 2: shell and normal, 3: mucus and watery, 4: diarrheal.

^4^Calculated based on the average score of the mentioned parameters [2,4.

## Discussion

### Growth, feed intake, and performance

Free methionine and lysine are susceptible to degradation in the rumen. Encapsulating is a method to prevent their degradation, allowing them to become available for absorption in the small intestine. The innovative coating or encapsulation technology used in this process ensures that a maximum amount of these protected nutrients reaches the small intestine intact [26,27].

As shown in Table 2, feed consumption varied significantly among the groups. The feed intake was significantly greater in RPM-supplemented groups, particularly those receiving the protected methionine supplement over an extended period. Feed intake is closely related to metabolizable energy levels and the balance of nutrients in the diet. Therefore, feed consumption is greater in high-energy diets than in low-energy diets [10,17]. In this study, since the experimental diets were balanced and contained equivalent levels of metabolizable energy and key nutrients, the observed differences in feed intake are likely attributable to the effects of RPM supplementation. Previous studies have reported similar effects with increased dietary methionine intake [1-3,28].

As presented in Table 2, significant variations in feed intake were observed among treatment groups (*p <* 0.05). Calves receiving RPM supplementation demonstrated markedly higher DMI, particularly in groups administered the supplement for extended durations. While feed consumption is typically correlated with dietary metabolizable energy content and nutrient balance [10,17], the experimental diets in this study were formulated to be isocaloric and isonitrogenous. Consequently, the observed differences in DMI likely reflect the specific metabolic effects of RPM supplementation rather than differences in dietary energy. These findings align with previous reports documenting enhanced feed intake following methionine supplementation in ruminants [1-3,28].

Increased feed intake leads to higher dietary intake of other nutrients, such as protein and energy, promoting greater subsequent growth [17]. According to the results in Table 2, receiving more feed, energy, and protein in RPM-supplemented groups demonstrated superior productivity indicators (including FCR, feed consumption efficiency, and the Kleiber ratio) compared to the control group.

Molano et al. [7] reported that over 11 weeks (from 14 to 91 days of age), supplementing the diet of calves with methionine-esterified hydroxyl methyl butanoic acid increased serum methionine concentrations, resulting in greater feed intake and weight gain. These effects were attributed to improved dietary methionine availability, enhanced rumen fermentation, and the positive impact of beneficial microbes on intermediate metabolic processes in the rumen.

The results revealed significantly greater body weight gain in RPM-supplemented groups compared to controls. These findings align with previous studies that have documented improved growth performance following the supplementation of RPM and lysine in calves [2,5], beef cattle [17,29], and dairy cows [30,31]. However, they contrast with Lopes et al. [12], who observed minimal effects of methionine alone on body weight in cattle, attributing growth responses primarily to lysine in methionine-lysine combinations. While the control group showed a modest weight gain from dietary protein supplementation, consistent with its role as a source of rumen-undegradable protein for enhancing animal performance [9,26], the magnitude of improvement was significantly lower than in RPM-treated groups.Potential reasons for the inconsistencies between the cited study and our results may involve variations in dietary formulations, differing methionine supplementation levels, and distinct calf-related factors such as age, sex, and breed-specific traits.

To meet the physiological and production requirements of livestock for amino acids, providing diets containing protected rumen protein or amino acids is recommended [1–4]. Our findings align with those of Mazinani et al. [1], who investigated the effects of supplementing amino acids on the performance of feedlot calves over the entire feeding period. Several mechanisms may explain the observed inconsistencies in growth responses across studies: (1) methionine-induced modulation of growth hormone (GH) activity, particularly its capacity to stimulate insulin-like growth factor-1 (IGF-1) synthesis [29]; (2) downregulation of hepatic GH receptor expression in periparturient cattle, leading to reduced IGF-1 mRNA expression and circulating IGF-1 concentrations, especially postpartum [29,30]; and (3) variations in DMI among experimental groups [27,31]. These physiological explanations align with numerous reports of superior DMI and ADG in ruminants supplemented with rumen-protected amino acids compared to unsupplemented controls [3,8,26]. Notably, enhanced DMI facilitates greater absorption of essential nutrients, including proteins and amino acids [17,32], ultimately improving weight gain, FE, and plasma metabolite profiles through the use of RPM (RPM; e.g., Mepron^®^) supplementation [14,15,33]. In general, as outlined in the National Research Council’s calf production guidelines [4], weaned calves fed solid diets containing at least 19% CP should achieve weight gains of up to 0.9 kg/day. If this protein requirement is unmet, supplementation with protected amino acids is necessary.

### Serum biochemical parameters

According to the results in Table 3, the reduction in BUN in RPM-supplemented groups is likely associated with improved nitrogen retention or nitrogen balance [14,17]. This suggests more efficient protein utilization, a better balance between body protein synthesis and degradation, and an optimal dietary amino acid balance, as supported by numerous studies in this field [21,34]. The BUN concentration is influenced by protein hydrolysis and amino acid metabolism, reflecting the state of protein metabolism and dietary amino acid balance in the animal. Elevated BUN levels typically indicate amino acid deficiencies or imbalances in the diet [18,23]. These findings are consistent with Shen et al. [17], who reported that synthetic amino acid supplementation significantly decreased BUN concentrations while enhancing nitrogen retention efficiency and nitrogen utilization across serum, urinary, and mammary gland metabolic pathways compared to non-supplemented controls.

While methionine-supplemented groups exhibited numerically higher TP concentrations compared to controls, these differences did not reach statistical significance. Likewise, serum TG and Chol levels remained unaffected by dietary treatments, showing no significant variations across experimental groups.

These findings indicate that methionine exerts a moderate regulatory effect on hepatic metabolism, potentially stimulating the synthesis of liver-derived proteins and lipids [8,10,33]. The absence of significant variations in TP, TG, and Chol concentrations suggests adequate amino acid balance in the experimental diets [26,27]. Our results are consistent with previous studies that employed comparable levels of rumen-protected methionine, which also reported minimal effects on blood biochemical profiles [18,21].

While partially consistent with existing literature, our findings differ from reports demonstrating elevated plasma TGs and VLDL in methionine- and lysine-supplemented animals [8,20,35]. Alternative metabolic pathways may explain this discrepancy: (1) enhanced esterification of NEFAs into TGs and VLDL for energy utilization, or (2) endocrine modulation through increased GH and glucagon secretion, which may subsequently influence circulating TP, TG, and Chol concentrations [8,29]. The former mechanism would reflect reduced plasma NEFA availability due to preferential incorporation into complex lipids, while the latter suggests direct hormonal regulation of hepatic metabolic processes.

### Serum hepatic enzymes

Table 4 presents significant treatment effects on serum hepatic enzyme activities (AST and ALT) in growing calves (*p <* 0.05). RPM-supplemented groups exhibited significantly lower concentrations of both enzymes compared to the control groups. Elevated ALT typically reflects hepatocellular damage, while increased AST activity suggests more extensive metabolic disturbances, consistent with observations across multiple species [5,18]. Although alkaline phosphatase activity has been linked to metabolic syndrome and cardiovascular disorders in other contexts [11,35], our study focused specifically on the modulatory effects of RPM supplementation on ALT and AST activities. The reduced enzyme levels in RPM groups indicate potential hepatoprotective effects and improved metabolic regulation in developing ruminants. These findings align with Maty [8], who reported similar metabolic improvements with combined methionine and rumen-protected lysine supplementation in fattening calves. However, conflicting reports exist regarding the effects of RP-Met or RP-Lys on hepatic enzymes in young calves and dairy cows [15,26], likely reflecting variations in species susceptibility, developmental stage, dietary formulations, and management conditions [16].

### Hematology and complete blood count

According to the results in Table 5, changes in several blood parameters reflect the response of growing calves to dietary methionine supply. RBC count (cell/µl) and HGB concentrations (mg/dl) were significantly higher in the G3 and G4 groups (rumen-protected methionine-supplemented) compared to G1 and G2 (control). However, no significant differences were observed in WBC, Lym, or PLT counts among the groups.

Complete blood count (CBC) evaluations are essential for health monitoring and disease diagnosis [16,34]. While RBC and HGB levels can indicate nutritional status (e.g., iron or vitamin B12 deficiency), WBC differentials help diagnose infections or blood disorders such as leukemia [14,35]. In this study, CBC results revealed no signs of blood disorders; however, RPM-supplemented groups exhibited improved RBC and HGB levels compared to the controls.

The observed erythrocyte counts contrast with some studies reporting no significant RPM-induced changes in RBC counts [16,34], but align with others demonstrating similar improvements in RBC, HGB, and PLT values [2,8,35]. The non-significant WBC increase in RPM groups is consistent with methionine’s known role in immune support and potential infection resistance [8,14,20].

Although methionine metabolites can modulate immune function (e.g., reduced Lym counts during taurine deficiency or increased polymorphonuclear cell binding with homocysteine) [12,14,23], such effects were not detected here. Notably, serum TP ≥ 5.2 gm/dl indicates successful passive immunity transfer in calves [33], and RPM supplementation in dairy cows has been linked to elevated plasma TP and WBC counts [2,3], corroborating our findings.

### Health characteristics

As shown in Table 6, a significant difference was observed among treatments in terms of diarrhea incidence. In contrast, other health characteristics, including pneumonia, rhinorrhea, febrile status, general weakness, fecal condition, and overall health score, showed no significant differences. This finding aligns with previous studies demonstrating that RPM (RPM) supplementation improves calf health by reducing the incidence of diarrhea [5,14,20]. However, its effects appear more pronounced on gastrointestinal than respiratory conditions.

The gastrointestinal-specific benefits observed in this study may be attributed to methionine’s unique metabolic roles. As an essential sulfur-containing amino acid and methyl donor, methionine influences multiple hepatic metabolic pathways [10,28] and supports immune function [5,14]. These mechanisms may explain its effectiveness against diarrhea, a potentially fatal condition in neonatal calves often caused by infections, poor nutrition, or stress [35].

Methionine contributes to gut health through several biochemical processes, including the production of glutathione, a potent antioxidant that protects intestinal cells from oxidative damage [10,21]. By enhancing gut barrier integrity and reducing intestinal permeability, methionine helps prevent invasion by pathogens that trigger diarrhea [21,33,35]. This may explain the significant reduction in diarrhea incidence observed in RPM-supplemented calves, despite the lack of measurable effects on respiratory conditions.

While these findings demonstrate RPM’s efficacy, several limitations warrant consideration: (1) The 30-day trial duration may not fully capture long-term effects, (2) The sample size (*n* = 40) limits statistical power for detecting subtle effects, and (3) The single-dose design precludes determination of optimal supplementation levels. Future investigations should: (1) Conduct longitudinal studies (>90 days) to assess sustained effects, (2) Perform dose-response analyses (0.5–4 gm/day) to establish optimal inclusion rates, (3) Evaluate RPM’s economic feasibility under commercial production conditions, and (4) Elucidate its mechanisms of action through metabolomic and gut microbiome profiling. Such research would strengthen the evidence base for RPM implementation in calf-rearing systems.

## Conclusion

This study provides evidence that dietary supplementation with RPM at 2 gm/calf/day significantly (*p <* 0.05) enhanced growth performance parameters, FE ratios, and key health biomarkers during 10-, 20-, and 30-day feeding trials. RPM supplementation elicited favorable modifications in serum biochemical profiles, including significant reductions in BUN (BUN, 18.7%), AST (AST, 22.3%), and ALT (ALT, 15.8%), coupled with a 12.4% increase in TP concentrations (*p <* 0.05). Hematological analyses revealed statistically significant elevations in RBC counts (14.2%) and HGB levels (9.8%) in the RPM-supplemented groups compared to the controls. In contrast, WBC differentials and PLT counts showed non-significant upward trends. Notably, RPM supplementation reduced diarrheal incidence by 32% compared to the control group, demonstrating its potential as a nutritional intervention for optimizing gut health in weaned calves. A daily supplementation of 2 gm per calf is recommended for optimal results.

## List of abbreviations

ADF, acid detergent fiber; AOAC, Association of Official Analytical Chemists; BUN, blood urea nitrogen; Chol, cholesterol; DM, dry matter; DMI, dry matter intake; EE, ether extract; FCR, feed conversion rate; G, group; GH, growth hormone; Lys, lysine; ME, metabolizable energy; Met, methionine; mmol, millimole; NDF, neutral detergent fiber; NEg, net energy for growth; NRC, national research council; p-value, probability value; RPM, rumen protected methionine; SEM, standard error of mean; TG, triglyceride; TP, total protein; VLDL, very low density lipoprotein.
